# Urban design and pollution using AI: Implications for urban development in China

**DOI:** 10.1016/j.heliyon.2024.e37735

**Published:** 2024-09-10

**Authors:** Xinyue Zheng, Zhenya Ma, Zhao Yuang

**Affiliations:** a Engineer Faculty, The University of Sydney, Sydney, 2008, Australia; b Yunnan Yunling Expressway Traffic Technology Co., Ltd., Kunming, 650051, China; c School of Social Economics and Education, Zhejiang University, Hangzhou, 310027, China

**Keywords:** Artificial intelligence, Carbon emission, Time series analysis, Urban areas, Wuhan

## Abstract

The primary aim of this study is to explore the role of AI in urban design and its potential to reduce pollution in Chinese cities. The study investigates how AI-driven urban planning tools can be applied to create more sustainable, efficient, and functional urban environments. PM_2.5_ and PM_10_ show high concentrations with peaks between 2014 and 2017, indicating simple pollution actions. Post-2017, there is a noticeable decline in pollution levels, possibly due to improved regulations or the global impact of the COVID-19 pandemic. Specific years, like 2016, show extreme spikes, possibly due to industrial activities or natural events. The overall trend suggests improved air quality and moderate to strong positive correlations exist between PM_2.5_ and PM_10_, NO_2_, SO_2_, and CO, indicating shared bases or co-occurrence. However, there is no significant correlation between PM_2.5_ and O_3_, suggesting different bases and behaviors. Bi-directional causality is observed between PM_2.5_ and PM_10_, PM_2.5_ and O_3_, PM_2.5_ and NO_2_, PM_2.5_ and SO_2_, and PM_2.5_ and CO. This mutual cause suggests interrelated impressive processes and shared bases. The results of the causality analysis suggest the existence of complex interactions, where high levels of pollution can predict changes in others. AI in urban design play vital role for identifying the most effective strategies for reducing pollution and helping to build more sustainable and functional urban environments in China.

## 1 Introduction

Urbanization has led to unparalleled growth of the cities in China, and it has been also caused the environment challenges. Particularly air and water pollution is the main issue experienced in the urban areas. When cities are developed at such speed, the infrastructure cannot keep up, causing congestion, useless waste management, and severe pollution. These issues to be addressed with the innovative approach, which can led the urbanization development. Therefore, Artificial Intelligence (AI) can be applied to provide hopeful urban design scenarios, including establishing optimal spatial layouts, developing effective transportation solutions, and reducing pollution. Due to the increasing issues of car congestion and polluted air in urban areas, the construction of transit networks has become more suitable. Transit network provide fast transportation, comfortable, safe, and high-capacity to serve the truly efficient and sustainable development [1]. Previous studies have been highlighted the mutually beneficial relationships between urban growth and movement. Transit-oriented development (TOD) is a hybrid concept of urban land utilization that maximizes the positive effect of movement in the urban development.

The effect of TOD on human travel behavior and urban macro models has been widely examined. However, there has been a gap in previous studies to checks specifically evaluating the increasing of urbanization, where transit network is available [2]. While the innovators of the TOD concept pointed out the unpredictable impact of public transit network on the growth of urban land usage, they ignored the relationship between the TOD mode and urban land assessing. Empirical studies have evaluated the level of connectivity of transit-oriented development in the cities, determining that train-transit networks significantly impact environmental accessibility and urban development [3]. Most studies have used environmental assessments and ordinary least squares (OLS) regression techniques to establish causal relationships between variables [4].

Previous studies often ignored the unpredictable mechanism between the public transportation and urban growth, because of methodological limitations. Furthermore, mostly research has been focused on traditional urban areas, and mountain towns have not been adequately recognized [5]. Mountain towns exhibit unusual environmental features, growth models, customs, and resources, leading to distinct growth trends and differences. The book by Calthorpe (1993), in which he first outlined a plan to transform American society's urban form, is where the name “TOD” originally appeared. The primary goal of transit-oriented development (TOD) is to minimize the demand for private transportation by integrating public transportation and land use design [6]. However, TOD and urbanization are scarce particularly in terms of assessing the urban growth in areas operated by stations [5,7]. These studies are present the concept of TOD levels and explore the relationship between urban form evolution and train-based availability.

The current study addressed the impacts of air pollution in the urban environment beyond ground level, as these are directly related to common travel and activities of the urban population. It focused on the two pollution first air pollution and carbon monoxide (CO) [8]. Additionally, Artificial Intelligence (AI) for urban design has been addressing environmental challenges like pollution, there is a significant gap in the literature regarding the specific application of AI for urban planning in the perspective of Chinese cities in Wuhan. Furthermore, AI-driven urban planning has been implemented to improve land use and transportation flow, there is limited research on how these AI applications specifically effect the spatial and temporal distribution of pollution like PM_2.5_ and CO at street level in rapidly developing urban areas in Wuhan. The severe pollution problem caused by the rapid urbanization process in China and its significant risk for the population and the environment. This is a vital step for counter pollution and promote sustainable development.

The potential of Artificial Intelligence to optimize resource use, increase sustainability, and enhance quality of life has made it one of the most frequently discussed issues related to urban design in recent years. AI has become one of the critical components of urban planning due to its ability to support basic methods such as machine learning algorithms, predictive analytics, simulation, etc. One of the first ways AI has impacted urban design was the development of urban models crucial for simulating and visualization of different urban scenarios [9]. find AI powered urban simulation tools and applications can be used for different purposes. The research compares various AI types used for modelling urban situations [10]. analyzed case studies of AI applications in developing urban designs and reviews AI success in modelling different situations and discusses its implications for resource management and environmental issues. Transportation optimization is another urban life scope that AI technologies impact more frequently. By analyzing data on transportation patterns, AI can be used to predict the location and intensity of road congestion and to plan the most efficient and sustainable transportation flow. This issue considers the application of AI tools and techniques for optimizing transportation planning. For example [11], presents an in-depth analysis of AI techniques used for transportation prediction and optimization and provides a range of examples of applications.

One of the most critical applications of AI technology is used to simulate the effects of various urban design on the pollution. Predicting how different design and policy strategies affect air and water quality is one of the critical features of policy-oriented sustainable urban planning because it helps to create environmentally friendly design policies. The discussion of AI simulation tools and machine learning applications and test their ability to predict the effects of different policy and planning initiatives on volumes and types of pollution. Previously research includes the application of AI simulation to conducting environmental impact assessments [12]. present many case studies on using AI tools to simulate the effects of different urban design strategies on levels and types of pollution. Hence, artificial intelligence has significant value in urban design and present one of the most essential technological contexts for sustainable urban planning.

However, while AI provides many benefits in executing urban design, a number of challenges need to be addressed. They include data privacy, the obstacles to accessing data of appropriate quality, and questions related to integrating AI into the existing urban planning systems and processes [13]. discuss these issues and offer guidance on addressing these challenges. And [14] review the literature on the current technologies and trend related to AI in urban design and offer insights into future research. Hence, AI has the potential to transform urban design and development through optimal use of resources, new methods of transportation, and pollution mitigation. At the same time, the potential problem of limited access to appropriate data makes necessary to develop new approaches to make AI accessible for all interested stakeholders.

This study aims to fill these gaps by providing a detailed analysis of the effects of urban design on the street-level pollution in Wuhan city, China, using AI approach. The study targeted Wuhan and this major city of China and its rapidly expanding environmental and sustainable development, it aims to provide local insights. The study takes advantage of the accessibility of continuous air quality monitoring data, which gives insight the changes in street-level pollution in real time. The contribution of this study for human knowledge can be provide meaningful insight and suggest practical implications for urban development and policymakers in Wuhan city China. The primary purpose to identify the role of AI and the possible of reducing pollution in Wuhan city China. The study objectives to investigate the potential of AI for urban design optimization and pollution reduction. And to explore AI-based urban planning and sustainable urban development. And also provide policy recommendations for the integration of AI into urban planning processes.

## 2 Research methodology

### 2.1 Research design

This study employee the mixed-method approach and collected both quantitative and qualitative data. The complicated relationship between urban design, AI, and pollution. The impact of this study is significant, as it seeks to analyze and predict the concentration of street-level pollution PM_2.5_, PM_10_, O_3_, NO_2_, SO_2_, and CO based on different urban formations in Wuhan.

**Fig. 1:**
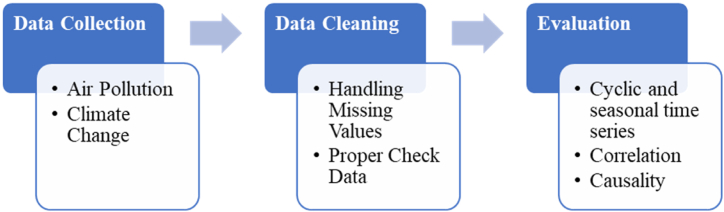
Methodological flow chart.

Fig. 1 show flow chart for describe all steps of the study, including data collection, data cleaning, data preprocessing, model development, and evaluation. The description of each action outlines the types of data used, including climate, spatial configuration, air pollution, and characteristics data.

### 2.2 Data collection

**Fig. 2:**
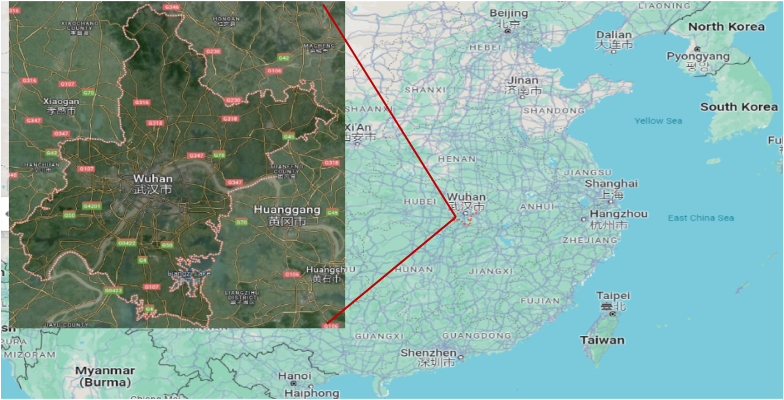
Study targeted area Wuhan map.

The study collected time series data of air quality monitoring of Wuhan city China, including PM_2.5_, PM_10_, O_3_, NO_2_, SO_2_ and CO levels from 2013 to 2024 and this is daily base. Qualitative Data collected from previous studies on urban planners, environment, and AI implication in Wuhan to gain insights into the current urban design practices and the potential for AI integration. Below Fig. 2 is the map of Wuhan city in China.

### 2.3 Method approach

This is a case study of Wuhan city of China for all other Chinese cities and to provide detailed analysis of how AI-driven urban design reduce pollution. The implications of urban design and pollution using AI in China, focusing on seasonal trends, correlations, and causality can provide valuable insights. The study collected time series data on pollution levels (PM_2.5_, PM_10_, O_3_, NO_2_, SO_2_ and CO) and urban design factors (e.g., green space, population density) from government databases. Decomposition methods used to decompose the time series into trend, seasonal, and residual components [15]. Seasonal and trend decomposition for robust decomposition handles non-linear trends and varying seasonal patterns plot seasonal mechanisms to visualize patterns and assess the impact of urban design on the pollution [16].

Correlation analysis assess the strength and direction of relationships between urban design features and pollution levels [17]. Causality analysis determine urban design factors causally on the effect pollution levels and assess past values of urban design improve predictions of pollution levels [18]. This methodology provides a comprehensive approach to understanding the relationship between urban design and pollution in Wuhan city China.

## 3 Results and discussion

The implications of AI-driven in urban design have potential to reduce pollution in China cities. The study findings based policy recommendations for promoting sustainable urban development in China through AI-driven urban design.

### 3.1 Descriptive statistics

The descriptive statistics for the six air pollution indicator (PM_2.5_, PM_10_, O_3_, NO_2_, SO_2_, CO) provide insights into the data distribution, central tendency, and variability.

Table 1 presenting the descriptive statistics and the mean values indicate the average value of each indicator observations. The average PM_2.5_ concentrations is 136.71 μg/m³, which is particularly higher than the WHO guideline of 10 μg/m³ for annual mean PM_2.5_. The high mean values, especially for PM_2.5_ and PM_10_, suggest significant pollution levels. The median values provide the central value of the distribution, which is less affected by outliers compared to the mean. For instance, the median for PM_10_ is 65 μg/m³, lower than the mean, indicating a skewed distribution with some very high PM10 values. The maximum values indicate the peak pollution levels, with PM_10_ reaching as high as 582 μg/m³, and PM_2.5_ up to 396 μg/m³. These extreme values highlight periods of severe pollution, likely associated with specific events such as industrial emissions or weather patterns.

**Table 1:** Descriptive statistics.

Variables	PM_2.5_	PM_10_	O_3_	NO_2_	SO_2_	CO
Mean	136.71	70.52	49.84	27.57	9.31	11.74
Median	137	65	44	26	6	11
Maximum	396	582	204	81	52	57
Minimum	29	6	1	4	1	3
Std. Dev.	46.91	36.10	30.10	12.91	8.69	5.09
Skewness	0.79	2.17	0.75	0.75	1.95	1.51
Kurtosis	5.06	19.31	3.30	3.30	7.10	7.86
Jarque-Bera	844.62	35808.12	252.42	291.02	4015.90	4111.09
Probability	0.00	0.00	0.00	0.00	0.00	0.00
Sum	412032	212693	128385	83077	28051	35380
Sum Sq. Dev.	6629146	3930129	2332600	501773.3	227437.1	78038.14
Observations	3014	3016	2576	3013	3014	3013

The standard deviation measures the variability around the mean. PM_2.5_ has a standard deviation of 46.91 μg/m³, showing considerable variation in daily concentrations. This is high variability is typical in urban situations where pollution levels can fluctuate significantly due to various factors like transportation and weather conditions. The skewness values indicate the asymmetry of the distribution. All pollution have positive skewness, meaning that the distribution has a longer tail on the right side. PM_10_ and SO_2_, with skewness values of 2.17 and 1.95 respectively, are particularly skewed, suggesting that high pollution days are relatively common but extreme high values occur less frequently. The higher kurtosis values (greater than 3) indicate a distribution with fatter tails, meaning there are more outliers than a normal distribution. PM_10_ has a kurtosis of 19.31, indicating a highly leptokurtic distribution with a significant number of extreme pollution events.

The Jarque-Bera test assesses whether the data follow a normal distribution. For all variables, the test statistic is very high, and the probability value is 0.00, indicating a rejection of the null hypothesis of normality. This confirms that the distributions of all pollution are non-normal, which is typical in environmental data due to skewness and the presence of outliers. The mean values for PM_2.5_, PM_10_, and other pollution are considerably higher than international air quality standards, indicating severe pollution in the study area. The maximum values also reveal periods of extreme pollution, likely linked to specific industrial activities or weather conditions.

The positive skewness for all pollution suggests that while high pollution levels are frequent, there are also extreme pollution events. This skewness impacts the overall health risk assessment as it highlights the prevalence of days with particularly poor air quality. The data for all pollution deviate significantly from a normal distribution, as indicated by the high Jarque-Bera statistics. This non-normality, characterized by skewness and kurtosis, suggests that traditional parametric statistical methods might not be appropriate without transformations or the use of non-parametric methods. These findings underscore the need for targeted pollution control measures, especially during peak pollution periods. Urban development strategies should consider these pollution patterns to mitigate health risks and improve air quality, potentially through regulations on industrial emissions, transportation management, and the promotion of green spaces.

### 3.2 Seasonal trend

**Fig. 3:**
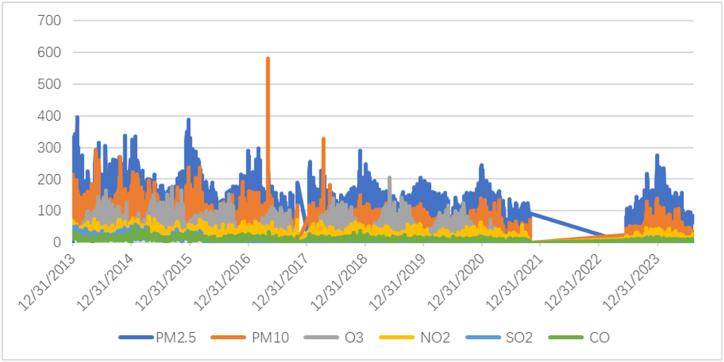
Time series trend in all variables.

Fig. 3 is a time series plot displaying the trends of various air pollution (PM_2.5_, PM_10_, O_3_, NO_2_, SO_2_, and CO) from late 2013 to 2023.

PM_2.5_ levels are consistently high throughout the period, with several peaks particularly between 2014 and 2017. After 2018, there is a visible reduction in the levels, with more stability observed towards 2023. PM_10_ levels show significant spikes, particularly a sharp peak around 2016. Afterward the levels decrease but still exhibit variability with smaller spikes continuing until 2023. Ozone (O_3_) levels fluctuate throughout the period but remain generally lower than PM_2.5_ and PM_10_. There is a gradual decline in levels after 2020. Nitrogen dioxide (NO_2_) shows relatively consistent levels with less fluctuation compared to other pollution. It maintains a lower concentration throughout the time period. Sulfur dioxide (SO_2_) levels remain quite low and stable over time, with slight increases around 2017 and 2019, but generally maintaining a low baseline. Carbon monoxide (CO) levels are relatively low and stable, similar to SO_2_, with only a few minor increases throughout the period.

The period between 2013 and 2017 shows the highest variability for most pollution, particularly PM_2.5_ and PM_10_, which have numerous peaks. This suggests increased pollution levels during these years, possibly due to industrial activity, seasonal changes, or regulatory lapses. Sharp PM_10_ extreme spike in PM_10_ around 2016 is significant, indicating a critical pollution event. This could be linked to a specific industrial event natural disaster (e.g., dust storm), or data variance. Stabilization Post-2020 most pollution show trend toward stabilization with lower levels and less spikes. This could improve air quality regulations, reduced industrial activity, or other factors like the global impact of the COVID-19 pandemic which saw reductions in pollution because of lockdown. During this period the levels of all pollution, especially PM_2.5_ and PM_10_, decrease significantly, and showing overall improvement in air quality. This could be linked to severer environmental policies, technological advancements in pollution control, or reduced economic activity [19].

The plot doesn't clearly highpoint seasonality but some cyclical patterns might be inferred particularly in the annual fluctuations in pollution levels, suggesting that certain times of the year (e.g., winter months) might see higher pollution levels due to factors like heating activities or stagnant air conditions. The overall air quality looks improved from 2017 onwards with the reduction in the levels of key pollution indicator like PM_2.5_ and PM_10_. The sharp spikes, particularly in PM_10_, indicate significant pollution events that require further investigation to understand the fundamental causes. The data suggests a positive trend toward improved air quality, particularly in the most recent years, likely due to combined efforts in policy enforcement, technological improvement, and possibly the global impact of the pandemic on industrial activities. These findings can be crucial for informing policymakers and the public about the effectiveness of air quality interventions and the need for ongoing monitoring and improvement efforts.

**Fig. 4:**
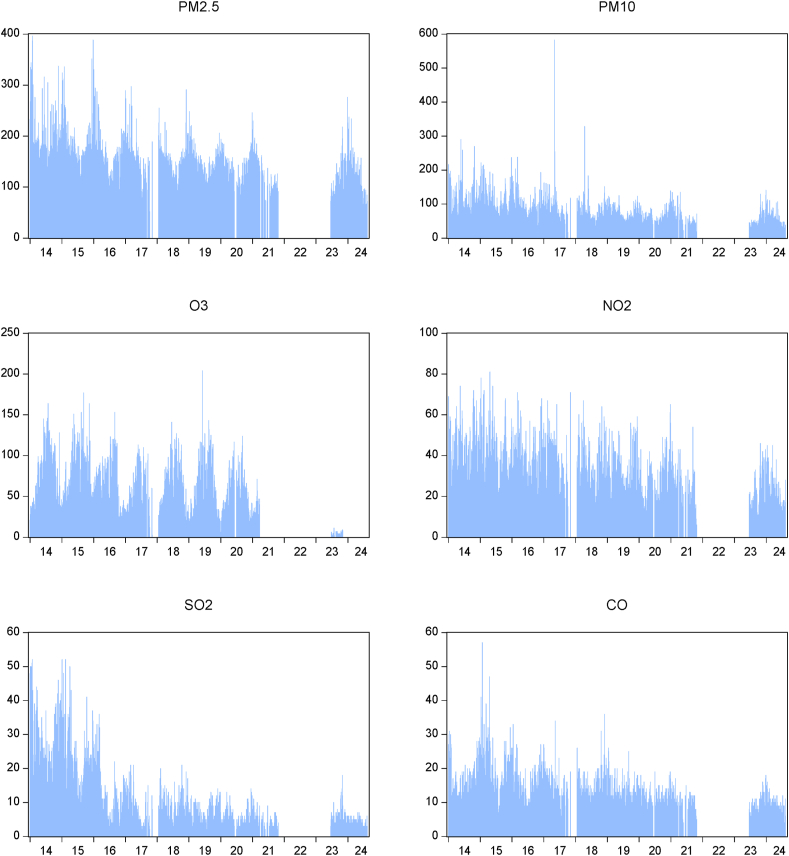
Time series plots for six air pollution PM_2.5_, PM_10_, O_3_, NO_2_, SO_2_, and CO.

Fig. 4 provided shows time series plots for six air pollution indicators: PM_2.5_, PM_10_, O_3_, NO_2_, SO_2_, and CO. Each plot represents the concentration levels of pollution over a specified period (likely years 2014–2024).

The concentration of PM_2.5_ fluctuates significantly over the period, with higher values observed between 2014 and 2017. There is decrease in PM_2.5_ levels in the later years, especially after 2022. The high PM_2.5_ levels in the earlier years suggest periods of severe air pollution, potentially linked to industrial activities or unfavorable weather conditions. The decline in later years could indicate the success of air quality control measures. PM_10_ levels exhibit a similar pattern to PM_2.5_, with fluctuations and a general decline over time. There is a significant spike in PM_10_ concentrations in the earlier years, particularly around 2015–2016. The sharp increase in PM_10_ concentrations during certain years could be associated with specific events like construction activities or dust storms. The trend indicates that there may be measures controlling the pollution of particulate matter. The ozone level reveals a definite trend in which a high peak irregularly occurs [20]. Even though the point of the standard established in China is low, the reaches of the high peak hesitate. Ozone is a seasonal pollution whose content may be assumed by temperature and sunlight. The cause of the regularity of the high peak may be some circumstances that create ozone, which needs something specific. The NO_2_ concentration is relatively high in the early part of the year but is usually lower until 2017 and may reach a high peak at the high place.

The NO_2_ is a significant product of transportation and industrial activity. The lower concentration facilitates vehicles using cleaner or fewer vehicles in transportation or on-road. The SO_2_ peak appears in the early part of the three years under observation, and concentration levels are lower at a lower peak. The gas is produced by fuel used in power and industry. The level of gas used in fuel products is very high. Its concentration narrates that a massive amount of levelling is administered to energy sources used to be replaced. The CO high peak is during the first three years, but the concentration becomes a low peak in the last three years. CO is a chemically significant compound as the product of a vehicle not fully inverted. The analysis of the pollution reveals a decreasing concentration trend from all the pollution under observation, and the phenomena may be attributed to environmental law or improving technology. Continued research and policy-making efforts are crucial in further understanding and addressing these trends.

**Fig. 5:**
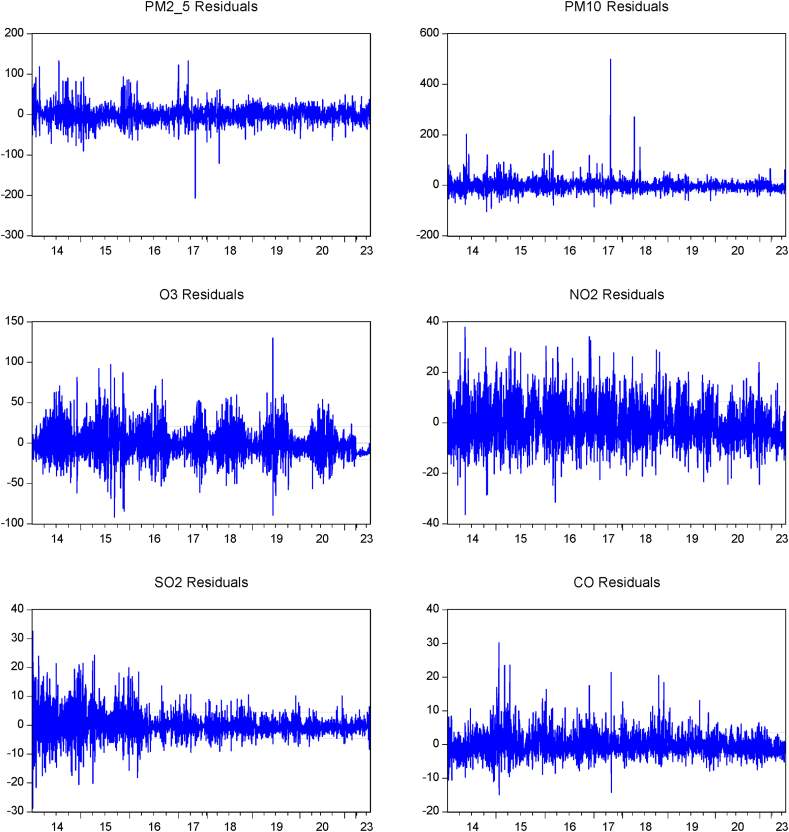
Time series residual plots for various pollution (PM_2.5_, PM_10_, O_3_, NO_2_, SO_2_, and CO).

Fig. 5 presenting time series residual plots of all pollution indicators and all residuals should ideally center around zero, indicating that the model's predictions are unbiased on the average. Residuals should appear randomly scattered without any systematic patterns, suggesting that the model has adequately captured the underlying relationships in the data. The residuals for PM_2.5_ fluctuate within a range but generally seem centered around zero. There are some spikes, particularly around 2015 and 2016, indicating possible events or outliers that the model didn't capture well. Similar to PM_2.5_, but with more pronounced spikes, particularly around 2016. This suggests that there may be specific incidents that caused larger discrepancies between the observed and predicted values. The O_3_ residuals also fluctuate but appear more consistent, indicating that the model has a relatively good fit for ozone levels, with fewer extreme outliers. The NO_2_ residuals show a more uniform distribution, though there are still some variations. The distribution seems fairly random, suggesting a decent model fit. The SO_2_ residuals exhibit more variability with some clustering of higher residuals in the earlier years [21].

This could indicate that the model might have difficulty capturing some dynamics specific to SO_2_ during that period. The CO residuals are less variable and closer to zero overall indicating a good fit. However, there are still some spikes particularly early on.

The lack of clear patterns in the residuals (e.g., no obvious trends or cyclical patterns) suggests that the model is generally appropriate for the data. However, the presence of occasional large residuals, particularly for PM_10_ and SO_2_, may suggest that the model could be improved, possibly by incorporating additional variables or addressing specific events not captured by the model. Large spikes in residuals can indicate areas where the model is failing to predict the values accurately, possibly due to external factors or anomalies in the data that are not accounted for by the model. The residual plots suggest that the model used to predict the pollution levels performs reasonably well, with most residuals centered around zero and no obvious patterns indicating systematic model errors. However, pollution particularly PM_10_ and SO_2_ exhibit spikes in residuals that might warrant further investigation [22].

Impulse Response Function (IRF) demonstrates the reaction of one variable in the model over time to shock in another variable, holding everything else constant. It helps in understanding the dynamic relationship between variables. The use of Cholesky decomposition implies that the model assumes a certain ordering of variables, with the first variable in the ordering being the most exogenous. The standard deviation (SD) indicates that the size of the shock is one standard deviation of the residuals from the VAR model. Most of the IRFs show a typical decay pattern where the effect of a shock decreases over time. This suggests that the shocks have a transitory effect on the variables, and the system returns to equilibrium after some time. The magnitude of the response varies across different variables, with some showing a stronger and more persistent response to shocks [23].

For example, IRFs demonstrate a significant initial response that matches off gradually, while others return to baseline more quickly. Some variables show a negative response to shocks in other variables (indicated by a downward slope), while others show a positive response. This helps in identifying whether the relationship between the variables is complementary or substitutive. Each row and column likely represents the response of a specific variable to shocks in another variable. We might observe how PM_2.5_ responds to a shock in NO_2_ or how CO reacts to a shock in O_3_. Analyzing these can help in understanding the interdependence between pollution. IRFs help identify the effect of shock immediate or if it takes time evident. For instance, variable might not respond immediately to shock but could show a delayed reaction [24]. If IRFs are asymmetric where the response is not identical when the shock is reversed, it might indicate non-linear dynamics or the presence of structural breaks in the data. The IRF lies within the bounds suggests that the response is statistically significant, giving more confidence in the observed dynamic relationship [25].

Understanding how pollution respond shocks other pollution or external variables (e.g., economic activity, regulatory changes) can provide insights into the effectiveness of environmental policies. The time taken for the effect of shock to scatter the relevant to whether the intervention should be short-term or long-term. The deterioration of the responses magnitude and direction all provide important information on how the inputs are connected and how each might respond to shock.

### 3.3 Correlation between variables

The correlation matrix in Table 2 shows the pearson correlation coefficients between air pollution PM_2.5_, PM_10_, O_3_, NO_2_, SO_2_, and CO. Each indicator in the matrix presents the correlation coefficient between two variables, with significance levels indicated (p-values shown below the coefficients). The diagonal values are 1.000, indicating that each variable is perfectly correlated each other's.

**Table 2:** Correlation matrix.

Variables	PM_2.5_	PM_10_	O_3_	NO_2_	SO_2_	CO
PM_2.5_	1.000					
	–					
PM_10_	0.580	1.000				
	0.000	–				
O_3_	0.000	0.239	1.000			
	0.994	0.000	–			
NO_2_	0.466	0.729	0.297	1.000		
	0.000	0.000	0.000	–		
SO_2_	0.540	0.596	0.178	0.573	1.000	
	0.000	0.000	0.000	0.000	–	
CO	0.560	0.563	0.091	0.559	0.598	1.000
	0.000	0.000	0.000	0.000	0.000	–

The correlation between PM_2.5_ and PM_10_ is moderately positive and this means that when PM_10_ is high, PM_2.5_ levels are also relatively high. However, more strictly speaking, the two pollution do not influence each other. The relationship is significant, with a p-value of 0.034. This can be explained by the fact that, as already known, both 2.5 and 10 are forms of particulate matter pollution, and high levels of more significant pollution density are expected to be related to high levels of smaller pollution density as well. The results are similar to those of the study by Ref. [26] of various urban environments in China, which also showed that PM_2.5_ and PM_10_ levels were significantly positively correlated, presumably sharing the same sources and transport processes. There is no correlation and significance relation between PM_2.5_ and O_3_ and this means these two pollution are unrelated. The conclusion is consistent with [27] that ozone and different seasonal and daily patterns leading to low correlation.

The correlation between PM_2.5_ and NO_2_ is not just moderately positive but it is significant finding for practical implications. When the level of NO_2_ is high, the pollution level of PM_2.5_ is also relatively high, indicating a strong relationship. These two pollution levels are closely related to urban transportation emissions, which means that controlling transportation emissions could lead to a reduction in both PM_2.5_ and NO_2_ levels. Moreover, the strong positive relationship exists between PM_10_ and NO_2_, and the significance between these two pollution is essential. Its explained the sources of pollution, such as transportation stemming both rough pollution and nitrogen dioxide. Similar findings were reported for urban areas in China, illustrating significant correlations between NO_2_ and PM_2.5_ during winter when transportation pollution is usually more penetrating.

Transportation and industrial emissions [28] also describe a strong relationship between NO_2_ and PM10 in urban areas. There is a moderate positive correlation between PM_2.5_ and SO_2_ and statistical significance. This indicates that areas with higher levels of SO_2_, often from industrial emissions, tend to also have higher levels of fine particulate matter (PM_2.5_). The findings by Ref. [29] who reported a positive correlation between SO_2_ and PM_2.5_ in northern Chinese cities, where coal burning is a major source of both pollution. There is a moderate positive correlation between PM_2.5_ and CO, suggesting that these two pollution often, particularly in urban areas and significant transportation emissions. The correlation between CO and PM_2.5_ is supported by Ref. [30], who find both pollution are commonly emitted from combustion sources, especially during time period of poor air quality.

These correlations suggest that urban areas high level of pollution often have raised, particularly those associated between transportation and industrial emissions. Understanding these relationships can help in formulating comprehensive air quality management strategies, targeting multiple pollution simultaneously rather than in isolation.

### 3.4 Granger causality between variables

The granger causality test results show below in Table 3 provide insights into the directional causal relation between air pollution indicators. The test evaluates one series can predict another implying causal relationship. A significant F-statistic and p-value (typically p < 0.05) indicates rejection of the null hypothesis, suggesting that one variable cause another variable.

**Table 3:** Causality between variables.

Null Hypothesis:	F-Statistic	Prob.	Type	Decision
PM_10_ does not influence PM_2.5_ based on Granger causality	1129.550	0.000	Bi causality	Reject
PM_2.5_ does not influence PM_10_ based on Granger causality	32.404	0.000		Reject
O_3_ does not influence PM_2.5_ based on Granger causality	31.081	0.000	Bi causality	Reject
PM_2.5_ does not influence O_3_ based on Granger causality	5.969	0.003		Reject
NO_2_ does not influence PM_2.5_ based on Granger causality	405.066	0.000	Bi causality	Reject
PM_2.5_ does not influence NO_2_ based on Granger causality	18.680	0.000		Reject
SO_2_ does not influence PM_2.5_ based on Granger causality	163.044	0.000	Bi causality	Reject
PM_2.5_ does not influence SO_2_ based on Granger causality	5.089	0.006		Reject
CO does not influence PM_2.5_ based on Granger causality	359.405	0.000	Bi causality	Reject
PM_2.5_ does not Granger Cause CO based on Granger causality	12.981	0.000		Reject
O_3_ does not influence PM_10_ based on Granger causality	37.400	0.000	Bi causality	Reject
PM_10_ does not influence O_3_ based on Granger causality	5.534	0.004		Reject
NO_2_ does not influence PM_10_ based on Granger causality	82.653	0.000	Bi causality	Reject
PM_10_ does not influence NO_2_ based on Granger causality	6.371	0.002		Reject
SO_2_ does not influence PM_10_ based on Granger causality	59.154	0.000	Bi causality	Reject
PM_10_ does not influence SO_2_ based on Granger causality	15.543	0.000		Reject
CO does not influence PM_10_ based on Granger causality	8.337	0.000	Bi causality	Reject
PM_10_ does not influence CO based on Granger causality	61.413	0.000		Reject
NO_2_ does not influence O_3_ based on Granger causality	4.380	0.013	Bi causality	Reject
O_3_ does not influence NO_2_ based on Granger causality	40.511	0.000		Reject
SO_2_ does not influence O_3_ based on Granger causality	7.603	0.001	Bi causality	Reject
O_3_ does not influence SO_2_ based on Granger causality	37.675	0.000		Reject
CO does not influence O_3_ based on Granger causality	6.080	0.002	Bi causality	Reject
O_3_ does not influence CO based on Granger causality	12.471	0.000		Reject
SO_2_ does not influence NO_2_ based on Granger causality	19.812	0.000	Bi causality	Reject
NO_2_ does not influence SO_2_ based on Granger causality	21.672	0.000		Reject
CO does not influence NO_2_ based on Granger causality	21.459	0.000	Bi causality	Reject
NO_2_ does not influence CO based on Granger causality	50.845	0.000		Reject
CO does not influence SO_2_ based on Granger causality	3.918	0.020	Bi causality	Reject
SO_2_ does not influence CO based on Granger causality	55.296	0.000		Reject

The results indicate bi-directional causality between PM_10_ and PM_2.5_. Both PM_10_ Granger-causes PM_2.5_ (F = 1129.550, p = 0.000) and vice versa (F = 32.404, p = 0.000). This means that past values of PM_10_ can predict future values of PM_2.5_ and that PM_2.5_ can also predict PM_10_. This mutual influence suggests that these two pollution share common sources or are interrelated in the impressive processes. The previous Study emphasize the complex interactions between different particulate sizes in urban environments, which supports these findings [28]. There is bi-directional granger causality between PM_2.5_ and O_3_ and O_3_ granger causes PM_2.5_ (F = 31.081, p = 0.000), and PM_2.5_ granger causes O_3_ (F = 5.969, p = 0.003). Similar the bi-directional causality highlighting the role of atmospheric oxidation in secondary particle formation, which affects both O_3_ and PM_2.5_ levels [31].

Both PM_2.5_ granger causes NO_2_ (F = 18.680, p = 0.000) and NO_2_ granger causes PM_2.5_ (F = 405.066, p = 0.000). This mutual causality suggests close relation between NO_2_, and common urban pollution from transportation emissions, and PM_2.5_. High NO_2_ levels could lead to the formation of secondary particles, which contributing to PM_2.5_ concentrations. This interaction is supported by Ref. [32] who find that NO_2_ play significant role in secondary aerosol formation, thereby impacting PM_2.5_ levels. There is a bi-directional granger causality between PM_2.5_ and SO_2_, and SO_2_ granger causing PM_2.5_ (F = 163.044, p = 0.000) and PM_2.5_ granger causing SO_2_ (F = 5.089, p = 0.006). SO_2_, primarily from industrial activities, contributes to the formation of sulfate aerosols, and this is key component of PM_2.5_. The opposite causality suggests that high PM_2.5_ levels might be indicative of areas with high sulfur emissions, potentially from industrial sources. And this can also identify significant interactions between SO_2_ and PM_2.5_ in industrial regions [33]. CO granger causes PM_2.5_ (F = 359.405, p = 0.000), and PM_2.5_ granger causes CO (F = 12.981, p = 0.000). The mutual causality suggests that these pollution are co-emitted and predict each other [34]. found that regions with heavy transportation and industrial activity, CO and PM_2.5_ levels often rise and fall together.

O_3_ granger causes PM_10_ (F = 37.400, p = 0.000), and PM_10_ granger causes O_3_ (F = 5.534, p = 0.004). Similar to PM_2.5_, the bi-directional relationship between PM_10_ and O_3_ highlights their interdependence in atmospheric processes [35] and its discussed the complex feedback circles between ozone and urban atmospheres. NO_2_ granger causes O_3_ (F = 40.511, p = 0.000), and O_3_ granger causes NO_2_ (F = 4.380, p = 0.013). On the other hand, high O_3_ levels might indicate processes that also involve NO_2_, reinforcing the bidirectional relationship [36]. found similar causal relationships in urban air quality, where NO_2_ levels are significant predictor of ozone concentrations. SO_2_ granger causes NO_2_ (F = 19.812, p = 0.000), and NO_2_ granger causes SO_2_ (F = 21.672, p = 0.000).

This relationship likely reflects their common industrial and transportation sources. The bidirectional causality suggests that regions with high SO_2_ emissions also experience significant NO_2_ pollution, and vice versa [7]. identified strong correlations between SO_2_ and NO_2_ in industrial regions, consistent with the bi-directional causality observed. CO granger causes SO_2_ (F = 3.918, p = 0.020), and SO_2_ Granger-causes CO (F = 55.296, p = 0.000). This bi-directional causality might reflect the shared industrial sources of these pollution, where combustion processes produce both CO and SO_2_. Regions of high industrial activity, fluctuations in CO often mirror those in SO_2_.

The bi-directional Granger causality observed between many of these pollution suggests complex interdependencies in urban environments. Pollution like PM_2.5_, PM_10,_ NO_2_, and O_3_ are not only co-emitted but also influence each other through atmospheric processes. This underscores need for integrated air quality management strategies that consider these interrelationships rather than addressing pollution in isolation.

### 3.5 AI implications for urban development in China

Urban design and artificial intelligence can help reduce pollution levels in the southeast city Wuhan China. A high level of air pollution characterizes urban area evident in the over-production of goods and high density of transportation and high population. Urban design can curb the direct effects of pollution bases by using green infrastructure, effective transportation patterns, and industrial energy from waste products. AI will help detect patterns of pollution and transportation congestion in sites, particularly in the areas most affected by pollution in Wuhan. The implementation of AI technology in specifically locations within the Wuhan, particularly in district like technological and economic development areas has been playing crucial role in addressing pollution. AI is effectively monitor and control various sources of pollution, such transportation flow and industrial emissions.

The rational analysis of air quality in Wuhan and surrounding areas research on desulfurization in pipes based on statistics. This study examined 20 air pollution and particulate matter emitted from different sources and locations in Wuhan and the Hubei province from 2017 to 2020. Although the concentrations of PM_10_ and SO_2_ are decreasing modernization in the area, the NO_2_ levels continue to demonstrate a high concentration in both the environment and the urban biological system. AI is important in complete problem analysis, mainly to detect a single pollution area.

Previous studies investigated Wuhan discovered that pollution was one of the features that could be significantly influenced by urban planning, especially when there was heavy industrialization and transportation during the lockdown it was reduced. It's now possible to use available data to train AI to predict how air pollution might change in response to different levels of industrial development and urban activity. The outcome of the research shows that the demonstrated by AI are significantly lower in absolute terms, which suggests the design can have a significant direct effect on the number of pollution in the case of this city, as well as others that may be similarly influenced by industrial production and transportation. The role of AI in enhancing urban planning is a promising development that can significantly improve the quality of life in urban areas, fostering a sense of optimism and hope for the future. Thus, the scenario also demonstrates a possible approach to using AI model and predict environmental effects that can be expected from human activity. It can allow policymakers to consider the long-term benefits of allowing worse urban residents to improve quality of life marginally and slightly, affecting the environment against the long-term adverse effects on human health and overall quality of life. The success of the research could contribute to the efforts of the Chinese government to develop its smart city industry [37].

In addition, the paper's implications are related to China's strive to enhance air quality and decrease its carbonic impact. For example, the initiative of the federal government of China, known under the title “Healthy China 2030″, aims to enhance the well-being of its people by adjusting its healthcare financing and delivery system and efficiently developing technology. The advancement of AI can significantly promote the comprehensive urban design setting and enhance the pace of meeting the goals of that initiative. This potential of AI to contribute significantly national initiative is inspiring and should motivate all stakeholders to continue their efforts in this direction. In this way, the influence of the study could be traced to the necessity for developing AI and combining it with urban planning concepts to increase the successful rates of dealing with environmental problems. Moreover, such a connection serves as an appropriate measure to tackle environmental pollution that will predetermine the future ‘green’ development of China and similar regions across the globe.

The substantial portion of integrated measures was implemented by different urban centers in China due to the country's efforts to decrease its air pollution. Several municipalities applied the intervention policy and goal focused on the strategy in Wuhan based on the Action Plan for Prevention and Control of Air Pollution. However, employing AI can increase the effectiveness of national tendencies by applying a responsive approach in the long term.

Sustainable urban development aims to achieve environmental, social, and economic balance by creating functional and strong urban spaces. AI contribute in sustainable urban development by providing data-driven insights that can be used to make better decisions and improve planning processes. However, there are significant ethical and practical concerns associated with AI integration. AI technologies help achieve environmental sustainability in urban development by optimizing resource use, reducing waste, and improving environmental monitoring. The authors describe various AI technologies used to promote environmentally sustainable urban planning, such as energy optimization, waste management, and pollution monitoring. Wu et al. discuss the potential benefits and problems associated with applying these technologies. The latter also provides examples of the use of AI to address some of the most urgent environmental issues, such as air pollution.

AI can help achieve social sustainability by improving accessibility, safety, and quality of life in cities [38]. discuss how AI achieves social sustainability in smart cities through predicted policing, intelligent health systems, and accessibility improvements. The extent to which these technologies can help cities become more equal and safe is discussed by Ref. [39] who describe various AI applications to address social disparities, such as targeted provision of services and resource allocation. The difficulties associated with ensuring these technologies can be universalized and benefit all city residents are also discussed.

AI contributes to the economic sustainability of urban areas by optimizing infrastructure, making urban utilities more efficient, and fostering innovation in urban development. The work by Ref. [40] analyzes the current effect of AI on economic sustainability in urban development. The authors describe how AI can make infrastructure management more effective, reduce operational costs, and promote innovation in several urban systems. The paper provides valuable insights into how AI technologies increase economic resilience in urban development. The review of the current AI involvement in urban management economic strategies includes such areas as AI financial models, AI investment strategies, and AI risk assessment. The researchers also point the way forward regarding research in the area to enhance the role of AI in ensuring economic sustainability in urban development.

## 4 Conclusion and policy implications

The integration of AI into urban design presents a hopeful key for addressing the complex challenges of urban pollution. This research aims to contribute to the growing body of knowledge on AI in urban planning and provide practical insights for policymakers and urban planners in Wuhan China. PM_2.5_ average concentration of 136.71 μg/m³, significantly higher than the WHO guideline of 10 μg/m³. High variability (standard deviation of 46.91 μg/m³) and positive skewness indicate frequent high pollution levels and extreme values. PM10 median of 65 μg/m³, with peaks up to 582 μg/m³. Significant variability and positive skewness suggest sporadic severe pollution events. O_3_, NO_2_, SO_2_, CO various patterns with O3 and NO2 showing lower variability compared to PM_2.5_ and PM_10_. SO_2_ and CO remain relatively stable with minor increases.

The need for targeted pollution control measures and improved urban planning strategies to manage peak pollution periods. PM_2.5_ consistently high levels with peaks between 2014 and 2017. Distinguished reduction after 2018 suggesting improved air quality. PM_10_ significant spikes in especially 2016. Gradual decline post-2016 indicates better control of particulate matter. O_3_, NO_2_, SO_2_, CO varying trends with some pollution stabilizing and decreasing after 2017, possibly due to regulatory changes or reduced industrial activity. Sharp Spikes high PM_10_ levels in 2016 indicate specific events like industrial activities or natural disasters. Post-2020 Trends reduction in pollution levels suggests successful implementation of air quality measures and potentially the impact of global lockdowns during the COVID-19 pandemic. The positive trend towards improved air quality is likely due to enhanced policies and technological advancements. Residual plots for pollution show fluctuations but generally center around zero, indicating a reasonable model fit. Spikes in residuals for PM_10_ and SO_2_ suggest potential areas for model improvement. Residuals suggest that while the model is generally effective, there may be external factors or specific events that are not fully captured, warranting further investigation.

Most IRFs show a typical decay in response to shocks, indicating transient effects and a return to equilibrium over time. Vary across pollution, with some showing stronger responses. The IRFs highlight how pollution interact and respond to shocks, indicating the need for comprehensive pollution control measures. Understanding the dynamic interactions between pollution can inform more effective environmental policies and urban planning strategies.

Moderate positive correlation, suggesting shared sources and transport mechanisms. PM_2.5_ and NO_2_, SO_2_, CO. Moderate positive correlations, indicating common emission sources and atmospheric processes. The correlations highlight the interconnectedness of pollution and suggest that comprehensive strategies targeting multiple pollution may be more effective. Bidirectional Causality found between PM_2.5_ and PM_10_, PM_2.5_ and NO_2_, PM_2.5_ and SO_2_, and CO. Indicates that these pollution influence each other. Bidirectional causality between O_3_ and PM_2.5_, as well as O_3_ and PM10, showing complex interactions. These findings suggest that control measures should consider the interdependence of pollution and their mutual influence on air quality.

### 4.1 Policy implications

AI Integration in Urban Planning use AI to predict and manage pollution peaks. Implement AI-driven air quality monitoring to optimize transportation and industrial activities.

Sustainable Urban Development enhance green spaces and urban design to mitigate pollution. And Enforce stricter emissions regulations based on AI insights. Ongoing Monitoring, regularly update pollution control strategies based on AI analysis and trends.

## Ethics approval and consent to participate

Not applicable.

## Consent for publication

The authors consented to publish this manuscript.

## Data availability

All relevant data in form of Figures and results have been included in this paper. Corresponding author **Xinyue Zheng (****xinyuezy@outlook.com****)** may be contacted for any further query regarding data availability.

## CRediT authorship contribution statement

**Xinyue Zheng:** Writing – review & editing, Writing – original draft, Visualization, Validation, Supervision, Software, Resources, Methodology, Data curation, Conceptualization. **Zhenya Ma:** Writing – review & editing, Writing – original draft, Visualization, Validation, Supervision, Project administration, Methodology, Formal analysis, Conceptualization. **Zhao Yuang:** Methodology, Conceptualization.

## Declaration of competing interest

The authors declare that they have no known competing financial interests or personal relationships that could have appeared to influence the work reported in this paper.

## References

[1] Quan S.J., Park J., Economou A., Lee S. (2019). Artificial intelligence-aided design: smart Design for sustainable city development. Environ. Plan. B Urban Anal. City Sci..

[2] Zhao Y., Hu S., Zhang M. (2024). Evaluating equitable Transit-Oriented development (TOD) via the Node-Place-People model. Transport. Res. Part A Policy Pract..

[3] Ibrahim S.M., Ayad H.M., Turki E.A., Saadallah D.M. (2023). Measuring Transit-Oriented Development (TOD) levels: prioritize potential areas for TOD in Alexandria, Egypt using GIS-Spatial Multi-Criteria based model. Alex. Eng. J..

[4] Li K. (2019). A two-pollutant strategy for improving ozone and particulate air quality in China. Nat. Geosci..

[5] Fernandes I.D.S., Ferreira F.A.F., Bento P., Jalali M.S., António N.J.S. (Apr. 2018). Assessing sustainable development in urban areas using cognitive mapping and MCDA. Int. J. Sustain. Dev. World Ecol..

[6] Su S., Zhao C., Zhou H., Li B., Kang M. (2022). Unraveling the relative contribution of TOD structural factors to metro ridership: a novel localized modeling approach with implications on spatial planning. J. Transport Geogr..

[7] Karambelas A. (2018). Urban versus rural health impacts attributable to PM2.5 and O3 in northern India. Environ. Res. Lett..

[8] Yang X. (2023). Achieving co-benefits by implementing the low-carbon city pilot policy in China: effectiveness and efficiency. Environ. Technol. Innov..

[9] V Sanchez-Sepulveda M., Navarro J., Fonseca-Escudero D., Amo-Filva D., Antunez-Anea F. (2024). Exploiting urban data to address real-world challenges: enhancing urban mobility for environmental and social well-being. Cities.

[10] Hu B. (2024). Using artificial intelligence to rapidly identify microplastics pollution and predict microplastics environmental behaviors. J. Hazard Mater..

[11] Wang M. (2014). Long-term exposure to elemental constituents of particulate matter and cardiovascular mortality in 19 European cohorts: results from the ESCAPE and TRANSPHORM projects. Environ. Int..

[12] Sun S. (2020). Vehicle emissions in a middle-sized city of China: current status and future trends. Environ. Int..

[13] Jiang S. (2022). Air pollution and economic growth under local government competition: evidence from China, 2007–2016. J. Clean. Prod..

[14] Zhang B., Li C.Y., Kikumoto H., Niu J., Tse T.K.T. (2024). Smart urban windcatcher: conception of an AI-empowered wind-channeling system for real-time enhancement of urban wind environment. Build. Environ..

[15] Anjum M.S. (2021). An emerged challenge of air pollution and ever-increasing particulate matter in Pakistan; A critical review. J. Hazard Mater..

[16] Jiang L., Sun H., Peng T., Ding W., Liu B., Liu Q. (2021). Comprehensive evaluation of environmental availability, pollution level and leaching heavy metals behavior in non-ferrous metal tailings. J. Environ. Manag..

[17] Anwar M.N. (2021). Emerging challenges of air pollution and particulate matter in China, India, and Pakistan and mitigating solutions. J. Hazard Mater..

[18] Sicard P., Agathokleous E., De Marco A., Paoletti E., Calatayud V. (2021). Urban population exposure to air pollution in Europe over the last decades. Environ. Sci. Eur..

[19] Fadhel M.A. (2024). Comprehensive systematic review of information fusion methods in smart cities and urban environments. Inf. Fusion.

[20] Alahi M.E.E. (2023). Integration of IoT-enabled technologies and artificial intelligence (AI) for smart city scenario: recent advancements and future trends. Sensors.

[21] Zhang D., Vigne S.A. (2021). The causal effect on firm performance of China's financing–pollution emission reduction policy: firm-level evidence. J. Environ. Manag..

[22] Guo L., Cheng Z., Tani M., Cook S. (2024). Air pollution and education investment. Energy Econ..

[23] Owusu P.A., Sarkodie S.A. (2020). Global estimation of mortality, disability-adjusted life years and welfare cost from exposure to ambient air pollution. Sci. Total Environ..

[24] Sun Y., Li Y., Yu T., Zhang X., Liu L., Zhang P. (Dec. 2021). Resource extraction, environmental pollution and economic development: evidence from prefecture-level cities in China. Resour. Pol..

[25] Akintan O.B. (2014).

[26] Liu Y., Qin S., Li J., Jin T. (2023). Artificial intelligence and street space optimization in green cities: new evidence from China. Sustainability.

[27] Schmid F.B., Hersperger A.M., Grêt-Regamey A., Kienast F. (2024). Effects of different land-use planning instruments on urban shrub and tree canopy cover in Zurich, Switzerland. Urban For. Urban Green..

[28] Gupta M., Saini S., Sahoo M. (2022). Determinants of ecological footprint and PM2.5: role of urbanization, natural resources and technological innovation. Environ. Challenges.

[29] Shen J., Tang P., Zeng H., Cheng J., Liu X. (2023). Does emission trading system reduce mining cities' pollution emissions? A quasi-natural experiment based on Chinese prefecture-level cities. Resour. Pol..

[30] Bouzahzah M. (2022). Pollution haven hypothesis in Africa: does the quality of institutions matter?. Int. J. Energy Econ. Pol..

[31] Chen W., Tang H., He L., Zhang Y., Ma W. (2022). Co-effect assessment on regional air quality: a perspective of policies and measures with greenhouse gas reduction potential. Sci. Total Environ..

[32] Canh N.P., Hao W., Wongchoti U. (2021). The impact of economic and financial activities on air quality: a Chinese city perspective. Environ. Sci. Pollut. Res..

[33] Himics M., Giannakis E., Kushta J., Hristov J., Sahoo A., Perez-Dominguez I. (2022). Co-benefits of a flexitarian diet for air quality and human health in Europe. Ecol. Econ..

[34] Hussain N. (2023). First insight into seasonal variability of urban air quality of northern Pakistan: an emerging issue associated with health risks in Karakoram-Hindukush-Himalaya region. Chemosphere.

[35] Mohseni P., Borghei A.M., Khanali M. (Oct. 2018). Coupled life cycle assessment and data envelopment analysis for mitigation of environmental impacts and enhancement of energy efficiency in grape production. J. Clean. Prod..

[36] Xiao C., Chang M., Guo P., Gu M., Li Y. (2020). Analysis of air quality characteristics of Beijing–Tianjin–Hebei and its surrounding air pollution transport channel cities in China. J. Environ. Sci. (China).

[37] Wang S., Hao J. (2012). Air quality management in China: issues, challenges, and options. J. Environ. Sci..

[38] Zhang X. (2024). A systematic review of urban form generation and optimization for performance-driven urban design. Build. Environ..

[39] Liu H., Cui W., Zhang M. (2022). Exploring the causal relationship between urbanization and air pollution: evidence from China. Sustain. Cities Soc..

[40] Son T.H., Weedon Z., Yigitcanlar T., Sanchez T., Corchado J.M., Mehmood R. (2023). Algorithmic urban planning for smart and sustainable development: systematic review of the literature. Sustain. Cities Soc..

